# Integrated network analysis and experimental validation identify CCNA2, CD44, and STAT1 as clinically relevant hub genes in oral squamous cell carcinoma

**DOI:** 10.3389/fmolb.2026.1748821

**Published:** 2026-02-09

**Authors:** Mingyang Bu, Yue Gu, Yuhan Meng, Yunyi Cui, Qianli Wu, Yali Kong, Haoyang Liu, Yi Chen, Jiajun Zhang, Lizhen Guo, Yakun Yang, Dongyang Huang, Zhe Ma

**Affiliations:** 1 Department of Preventive Dentistry, Hebei Key Laboratory of Stomatology, Hebei Clinical Research Center for Oral Diseases, School and Hospital of Stomatology, Hebei Medical University, Shijiazhuang, China; 2 Department of Pediatric Dentistry, Hebei Key Laboratory of Stomatology, Hebei Clinical Research Center for Oral Diseases, School and Hospital of Stomatology, Hebei Medical University, Shijiazhuang, China; 3 Department of Dentistry and Oral Surgery, Shinshu University School of Medicine, Matsumoto, Japan; 4 Department of Pharmacology, The Key Laboratory of Neural and Vascular Biology, Ministry of Education, The Key Laboratory of New Drug Pharmacology and Toxicology, Collaborative Innovation Center of Hebei Province for Mechanism, Diagnosis and Treatment of Neuropsychiatric Diseases, Hebei Medical University, Shijiazhuang, Hebei, China

**Keywords:** CCNA2, CD44, hub genes, oral squamous cell carcinoma, STAT1

## Abstract

Oral squamous cell carcinoma remains a major clinical challenge, with only marginal gains in long-term survival due to frequent recurrence, lymphatic dissemination and therapeutic resistance. Robust and biologically grounded molecular determinants are needed to improve tumor characterization and facilitate translational advances. Transcriptomic profiles from GSE30784, GSE9844 and TCGA-HNSC were integrated to identify reproducible dysregulated genes, followed by construction of a high-confidence PPI network for hub gene prioritization. Prognostic and diagnostic relevance was assessed using Kaplan–Meier, Cox regression and ROC analyses. Experimental validation was performed by immunohistochemistry in human OSCC tissues and by qPCR in OSCC cell lines. Cross-cohort integration yielded a reproducible set of DEGs, from which CCNA2, CD44 and STAT1 emerged as network-defined hub genes. All three genes were consistently upregulated across independent cohorts and showed significant prognostic and diagnostic associations. Functional enrichment indicated that these genes are embedded in proliferation-, adhesion- and stress-response–related signaling programs. Experimental assays further confirmed their elevated expression at both transcript and protein levels in OSCC tissues and cell models. CCNA2, CD44 and STAT1 constitute reproducible and functionally anchored hub genes in OSCC, carrying both diagnostic and prognostic implications. These findings substantially refine the molecular understanding of OSCC and provide rational entry points for subsequent mechanistic and translational investigations.

## 1 Introduction

Oral squamous cell carcinoma (OSCC) is the predominant subtype of head and neck malignancies and remains a leading cause of cancer-related mortality worldwide (Johnson et al., 2020). Despite advances in surgical intervention, radiotherapy and systemic therapies, long-term survival has improved only marginally over recent decades, owing mainly to frequent local recurrence, cervical lymph node metastasis and the emergence of therapeutic resistance (Dong et al., 2024; Kim and Ahn, 2024; Struckmeier et al., 2024). These limitations highlight the need to delineate molecular determinants that can refine disease stratification and provide biologically meaningful entry points for therapeutic development.

High-throughput transcriptomic studies have documented extensive gene dysregulation in OSCC (Guo et al., 2020; Li et al., 2023; Lohavanichbutr et al., 2012). However, many reported biomarkers lack reproducibility across independent cohorts, are not embedded within coherent regulatory networks, or remain unverified at the experimental level (de Morais et al., 2025; Hsu et al., 2020; Liang et al., 2004). Moreover, only a minority have been rigorously evaluated for prognostic relevance or mechanistic interpretability, leaving unresolved which alterations represent robust and clinically meaningful drivers rather than cohort-specific correlates.

To address these gaps, we employed an integrative framework combining multi-cohort differential expression analysis, network-based hub gene prioritization and cross-platform validation, followed by survival interrogation and experimental verification in human tissues and OSCC cell models. This strategy enables the identification of reproducible and network-supported candidate genes with diagnostic and prognostic utility, thereby refining the molecular framework of OSCC and establishing rational targets for downstream mechanistic and translational investigations.

## 2 Methods and materials

### 2.1 Public datasets and preprocessing

Transcriptomic datasets of OSCC were retrieved from the Gene Expression Omnibus (GEO) database (accessions GSE30784 and GSE9844), and RNA-seq profiles of OSCC samples were obtained from the Cancer Genome Atlas–Head and Neck Squamous Cell Carcinoma (TCGA-HNSC) cohort. GEO microarray data were normalized using the RMA algorithm, whereas TCGA raw counts were converted to TPM values (Bian et al., 2022). Probe identifiers were mapped to gene symbols according to platform annotations, and duplicate probes corresponding to the same gene were averaged. Only protein-coding genes detected in at least 80% of samples were retained for downstream analysis.

### 2.2 Differential expression analysis

Differentially expressed genes (DEGs) between tumor and normal tissues were identified using “limma” for microarray data and “DESeq2” for RNA-seq data. When applicable, batch effects were mitigated using “ComBat”. Genes with |log_2_FC| ≥ 1 and FDR < 0.05 were considered statistically significant. Volcano plots were generated to visualize the DEG distribution (Ritchie et al., 2015).

### 2.3 Protein–protein interaction network construction and hub gene identification

Overlapping DEGs from the two GEO datasets were submitted to STRING (confidence threshold = 0.9) to construct a protein–protein interaction (PPI) network (Zhang et al., 2022). The resulting network was imported into Cytoscape for topological assessment. Candidate hub genes were ranked using betweenness and degree centrality, and the intersection of the top 10 genes from both metrics was defined as the final hub gene set (Li et al., 2019). Sensitivity analysis was performed to assess the robustness of hub gene identification. Degree and betweenness centrality rankings were recalculated using different cutoff thresholds (top 5, top 10 and top 15 nodes). The overlap of candidate hub genes across thresholds and metrics was examined to evaluate the stability of network-based prioritization.

### 2.4 Gene set enrichment analysis

Gene set enrichment analysis (GSEA) was performed to explore pathways associated with hub gene expression using Hallmark, KEGG, and GO gene sets from MSigDB. All genes were ranked according to differential expression statistics, and the enrichment score was computed using a weighted Kolmogorov–Smirnov test. Pathways with FDR < 0.25 were considered significantly enriched (Subramanian et al., 2005).

### 2.5 TCGA validation and survival modeling

The expression patterns of hub genes were validated in the TCGA-HNSC OSCC subset. Prognostic relevance was assessed using Kaplan–Meier survival curves and Cox proportional hazards modeling (Rades et al., 2013). A nomogram incorporating CCNA2, CD44, and STAT1 was constructed based on the Cox model. Diagnostic performance of individual genes and the combined panel was evaluated using ROC analysis in GEO and TCGA cohorts (Long et al., 2022).

### 2.6 Human tissue specimens and immunohistochemistry

Thirty paired samples of primary OSCC tissues and matched non-tumorous oral mucosa (>2 cm from the tumor margin) were collected at the Hospital of Stomatology, Hebei Medical University. The study followed the Declaration of Helsinki and received approval from the institutional ethics committee (Approval No. 2025039); written informed consent was obtained from all participants.

FFPE sections were processed by standard deparaffinization, rehydration, antigen retrieval, and peroxidase/nonspecific blocking. Primary antibodies against Cyclin A2 (CCNA2) (Abcam, ab181973), Cluster of Differentiation 44 (CD44) (Cell Signaling Technology, #3570), and Signal Transducer and Activator of Transcription 1 (STAT1) (Proteintech, 10144-2-AP) were applied, followed by HRP-conjugated secondary antibodies and DAB detection with hematoxylin counterstaining. Two pathologists independently scored staining intensity under blinded conditions.

### 2.7 Cell culture

The human OSCC cell line CAL27 was obtained from the Cell Bank of the Chinese Academy of Sciences (CAS, Shanghai, China). Normal human oral keratinocytes (HOK) were purchased from ScienCell Research Laboratories (Carlsbad, CA, USA). All cell lines were authenticated by the suppliers and routinely tested to confirm the absence of *mycoplasma* contamination. Cells were cultured in DMEM supplemented with 10% fetal bovine serum and 1% penicillin–streptomycin at 37 °C in a humidified incubator with 5% CO_2_.

### 2.8 Quantitative PCR analysis

Total RNA was extracted using TRIzol reagent, and RNA quality was verified spectrophotometrically (A260/A280 = 1.8–2.0). One microgram of RNA was reverse-transcribed using a Takara PrimeScript™ RT reagent kit according to the manufacturer’s instructions. The resulting cDNA was diluted 1:5 with nuclease-free water prior to quantitative PCR amplification.

qPCR was performed using SYBR Green Master Mix on an ABI StepOnePlus system with a 20 µL reaction volume under the following cycling conditions: 95 °C for 30 s, followed by 40 cycles of 95 °C for 5 s and 60 °C for 30 s. Amplification specificity was confirmed by melting-curve analysis. GAPDH served as the internal reference, and relative expression was calculated using the 2^−^ΔΔCt method. All qPCR experiments were performed in triplicate. Data analysis and figure generation were conducted using GraphPad Prism.

The primer sequences used in this study were as follows:

CCNA2-F: 5′-AGCAGCCAGACATCACTCTC-3′
CCNA2-R: 5′-TGGTGCTGAAGGTAGGTGTC-3′
CD44-F: 5′-CTGCCGCTTTGCAGGTGTA-3′
CD44-R: 5′-GGTGCTATTGAAAGCCTTGC-3′
STAT1-F: 5′-GCTGGAAGATGGTGAAATTGAG-3′
STAT1-R: 5′-CATGATGGTTGTGCTGATGCT-3′
GAPDH-F: 5′-AGAAGGCTGGGGCTCATTTG-3′
GAPDH-R: 5′-AGGGGCCATCCACAGTCTTC-3′
CRNN-F: 5′-TGGCAGAGTGCAGAGGACTA-3′
CRNN-R: 5′-AGGTCAGGGAGGATGGAAGA-3′

### 2.9 Western blot analysis

Total protein was extracted from human oral tissue specimens and cultured cells using ice-cold RIPA lysis buffer (Beyotime, China) supplemented with a protease inhibitor cocktail (Beyotime, China), according to the manufacturer’s instructions. Lysates were incubated on ice for 30 min with intermittent vortexing and subsequently centrifuged at 12,000 × *g* for 15 min at 4 °C to remove insoluble debris.

Protein concentration was determined using a bicinchoninic acid (BCA) protein assay kit (Beyotime, China). Equal amounts of total protein (30 µg per lane) were separated by 10% SDS–PAGE and transferred onto polyvinylidene fluoride (PVDF) membranes (Millipore, USA). Membranes were blocked with 5% non-fat milk in Tris-buffered saline containing 0.1% Tween-20 (TBST) for 1 h at room temperature and then incubated overnight at 4 °C with primary antibodies against STAT1 (Proteintech, 10144-2-AP, 1:1,000 dilution) and β-actin (Proteintech, 60008-1-Ig, 1:5,000 dilution).

After washing with TBST, membranes were incubated with horseradish peroxidase (HRP)-conjugated secondary antibodies (Proteintech, 1:5,000 dilution) for 1 h at room temperature. Protein bands were visualized using enhanced chemiluminescence (ECL) reagents (Millipore, USA) and detected with a ChemiDoc imaging system (Bio-Rad, USA). Densitometric analysis was performed using ImageJ software (NIH, USA), and STAT1 protein expression levels were normalized to β-actin.

### 2.10 Statistical analysis

All statistical analyses were performed in R (version 4.2.0). Normality was assessed using the Shapiro–Wilk test. Comparisons between two groups were performed using Student’s t-test or the Mann–Whitney U test as appropriate. Correlations were assessed using Spearman’s rank test. Survival differences were evaluated by log-rank testing, and hazard ratios were estimated using Cox regression. ROC curves were generated using the “pROC” package. A two-sided P < 0.05 was considered statistically significant.

## 3 Results

### 3.1 Identification of robust DEGs in OSCC

**FIGURE 1:**
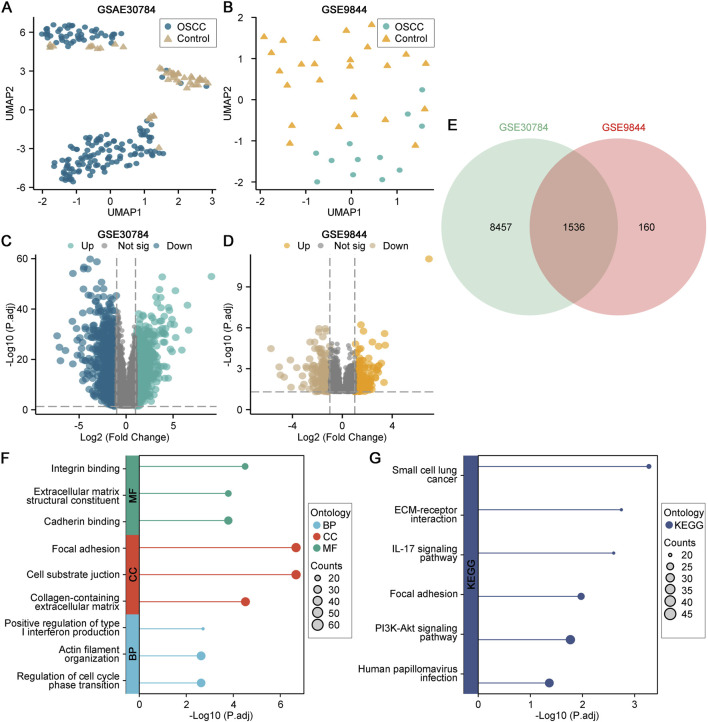
Transcriptomic profiling identifies reproducible DEGs in OSCC. **(A)** UMAP clustering of GSE30784 samples. **(B)** UMAP clustering of GSE9844 samples. **(C)** Volcano plot of DEGs in GSE30784. **(D)** Volcano plot of DEGs in GSE9844. **(E)** Overlap of DEGs between the two datasets. **(F)** GO enrichment of shared DEGs. **(G)** KEGG enrichment of shared DEGs.

To identify transcriptional alterations robustly associated with OSCC, we analyzed two independent GEO cohorts (GSE30784 and GSE9844). UMAP embedding revealed a clear separation between tumor and normal tissues in GSE30784, and a still discernible separation trend in GSE9844 despite partial overlap, indicating a disease-related divergence in global expression patterns (Figures 1A,B). Differential expression analysis further demonstrated widespread dysregulation in GSE30784, whereas GSE9844 showed a smaller but directionally consistent DEG spectrum. Volcano plots illustrated the distribution and significance of DEGs in both datasets (Figures 1C,D). To obtain high-confidence OSCC-related genes, differentially expressed genes shared between the two datasets were identified, as illustrated by the overlap analysis (Figure 1E). Functional enrichment analyses further revealed that these shared DEGs were predominantly involved in extracellular matrix organization, cell adhesion, and tumor-associated signaling pathways, including PI3K–Akt signaling and ECM–receptor interaction (Figures 1F–G).

### 3.2 Network-based prioritization and validation of hub genes in OSCC

Building on the shared DEGs identified above, we next sought to extract hub genes occupying central positions within OSCC-associated regulatory networks. A high-confidence PPI network was constructed in STRING (confidence score = 0.9), comprising 1,458 nodes and 1,294 edges with an average node degree of 1.78 and a clustering coefficient of 0.293 (Supplementary Figure S1). The number of observed edges markedly exceeded the random expectation (expected = 844), and the enrichment p-value < 1.0 × 10^−16^ indicated a non-random, functionally coordinated interaction architecture.

**FIGURE 2:**
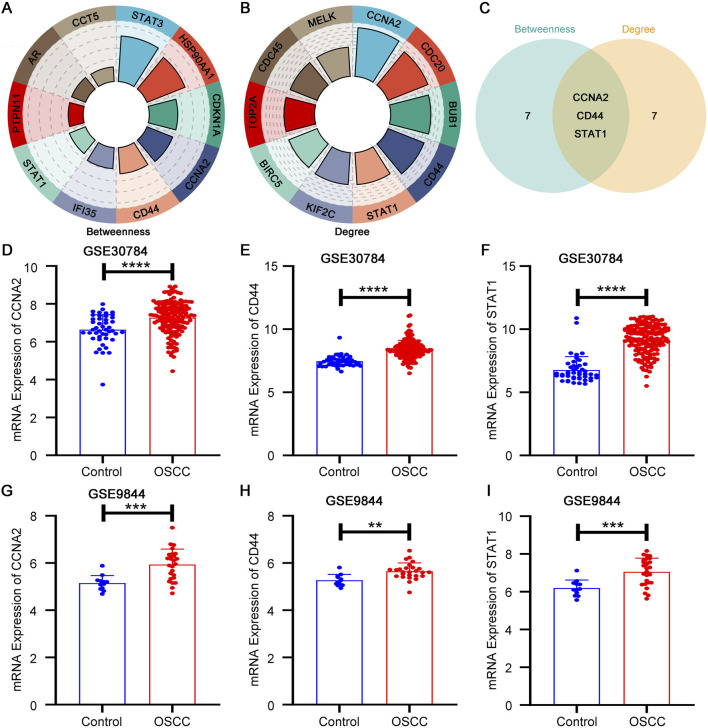
Network-based hub gene screening and expression validation in OSCC. **(A)** Top 10 hub candidates ranked by betweenness centrality. **(B)** Top 10 hub candidates ranked by degree centrality. **(C)** Intersection of two rankings identifying CCNA2, CD44, and STAT1 as shared hub genes. **(D–F)** Expression of CCNA2, CD44, and STAT1 in GSE30784. **(G–I)** Expression of CCNA2, CD44, and STAT1 in GSE9844.

Nodes were then ranked using two centrality metrics in Cytoscape. The top 10 candidates derived from betweenness and degree centrality were intersected, yielding CCNA2, CD44 and STAT1 as shared hub genes (Figures 2A–C). To validate these candidates, we examined their mRNA expression in two independent cohorts. CCNA2, CD44 and STAT1 were all significantly upregulated in OSCC tissues in GSE30784 and consistently elevated in GSE9844 (Figures 2D–I), supporting their stability across cohorts and endorsing them as credible hub regulators for downstream investigation.

To evaluate the robustness of hub gene selection, a sensitivity analysis was conducted by varying the ranking thresholds for degree and betweenness centrality (top 5, top 10 and top 15 nodes). CCNA2 and CD44 were consistently retained among the top-ranked genes across all thresholds and metrics, while STAT1 remained among the highest-ranked candidates in the majority of conditions (Supplementary Table S1). These findings indicate that the identification of CCNA2, CD44 and STAT1 is not driven by arbitrary cutoff selection, but reflects stable network centrality across multiple analytical settings.

### 3.3 Validation and characterization of hub genes in OSCC

**FIGURE 3:**
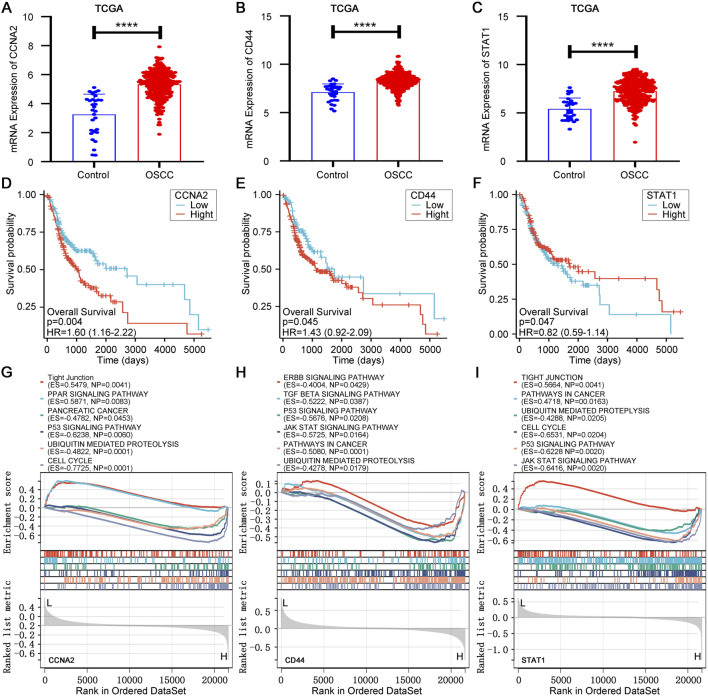
Validation and characterization of hub genes in TCGA cohort. **(A–C)** mRNA expression of CCNA2, CD44 and STAT1 in TCGA OSCC vs. normal tissues. **(D–F)** Kaplan–Meier overall survival curves stratified by CCNA2, CD44 and STAT1 expression. **(G–I)** GSEA enrichment plots for CCNA2, CD44 and STAT1.

To validate the robustness of the identified hub genes, we evaluated the expression patterns of CCNA2, CD44 and STAT1 in the TCGA OSCC subset. All three genes were significantly upregulated in tumor tissues compared with normal controls (Figures 3A–C), consistent with the GEO-based discovery. Kaplan-Meier analysis showed that high CCNA2 expression was associated with worse overall survival, and CD44 exhibited a similar adverse trend, whereas elevated STAT1 predicted a more favorable prognosis (Figures 3D–F).

To explore the functional context of these genes, we performed GSEA for each hub gene (Figures 3G–I). CCNA2 was enriched in pathways related to mitotic progression and protein degradation (e.g., Cell cycle, P53 signaling and ubiquitin-mediated proteolysis). CD44 was enriched in cancer-related signaling, tight junction disruption and JAK–STAT pathways, consistent with its roles in adhesion and malignant progression. STAT1 was enriched in P53, JAK–STAT and TGF-beta signaling, aligning with its involvement in immune surveillance and stress adaptation. Notably, all three genes converged on P53 signaling and ubiquitin-mediated proteolysis, suggesting potential cooperative modulation of stress response, genomic stability and protein turnover in OSCC.

To further explore whether the prognostic behavior of STAT1 may be influenced by the tumor immune microenvironment, immune infiltration analyses were performed using the TIMER and ESTIMATE algorithms in the TCGA-HNSC cohort. STAT1 expression exhibited significant positive correlations with multiple immune cell populations, including CD8^+^ T cells, CD4^+^ T cells, macrophages and dendritic cells, as well as with stromal enrichment scores (Supplementary Figure S2). These findings suggest that STAT1 expression in OSCC may partially reflect immune- and microenvironment-associated transcriptional programs.

### 3.4 Prognostic modeling and diagnostic discrimination based on CCNA2, CD44 and STAT1

**FIGURE 4:**
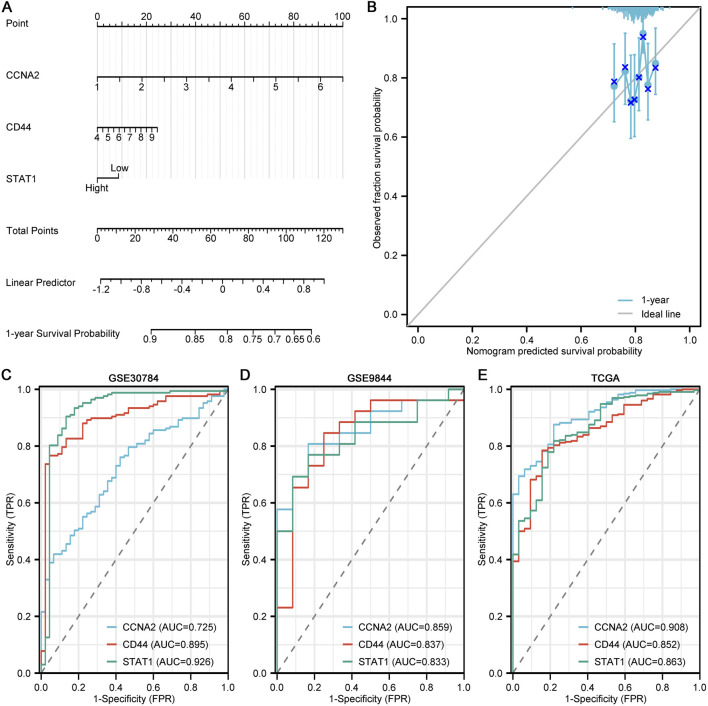
Prognostic nomogram and diagnostic performance of CCNA2, CD44 and STAT1. **(A)** Nomogram model based on CCNA2, CD44 and STAT1. **(B)** Calibration curve of the nomogram for 1-year survival prediction. **(C)** ROC curves of CCNA2, CD44 and STAT1 in GSE30784. **(D)** ROC curves of CCNA2, CD44 and STAT1 in GSE9844. **(E)** ROC curves of CCNA2, CD44 and STAT1 in TCGA.

Given the prognostic implications of CCNA2, CD44 and STAT1, we next assessed their utility in risk prediction and clinical discrimination. A nomogram incorporating all three genes was constructed using a Cox proportional hazards model, in which CCNA2 and CD44 contributed positively to the total risk score, whereas STAT1 exerted a negative contribution, consistent with their respective survival associations (Figure 4A). The calibration curve showed excellent agreement between predicted and observed 1-year survival probabilities (Figure 4B).

To further determine whether CCNA2, CD44 and STAT1 serve as independent prognostic factors, univariate and multivariate Cox proportional hazards regression analyses were performed using TCGA OSCC clinical data. Variables including age, sex and tumor stage were incorporated into the multivariate model. As shown in Supplementary Table S2, CD44 remained marginally associated with overall survival after adjustment, whereas CCNA2 and STAT1 did not retain independent prognostic significance. Age emerged as an independent clinical predictor of outcome, while sex lost significance after multivariable adjustment. These results indicate that the prognostic effects of the identified hub genes may be partially influenced by established clinicopathological factors, supporting their complementary rather than standalone prognostic value.

ROC analysis further demonstrated robust diagnostic performance across independent cohorts: in GSE30784, the AUCs for STAT1, CD44 and CCNA2 were all high; in GSE9844 they remained consistently discriminative; and in TCGA all three genes achieved AUC values above the generally accepted diagnostic threshold (Figures 4C–E). These findings indicate that this three-gene panel can reliably distinguish OSCC from normal tissues and holds stable prognostic and diagnostic utility across platforms.

### 3.5 Experimental validation of hub gene overexpression in OSCC

**FIGURE 5:**
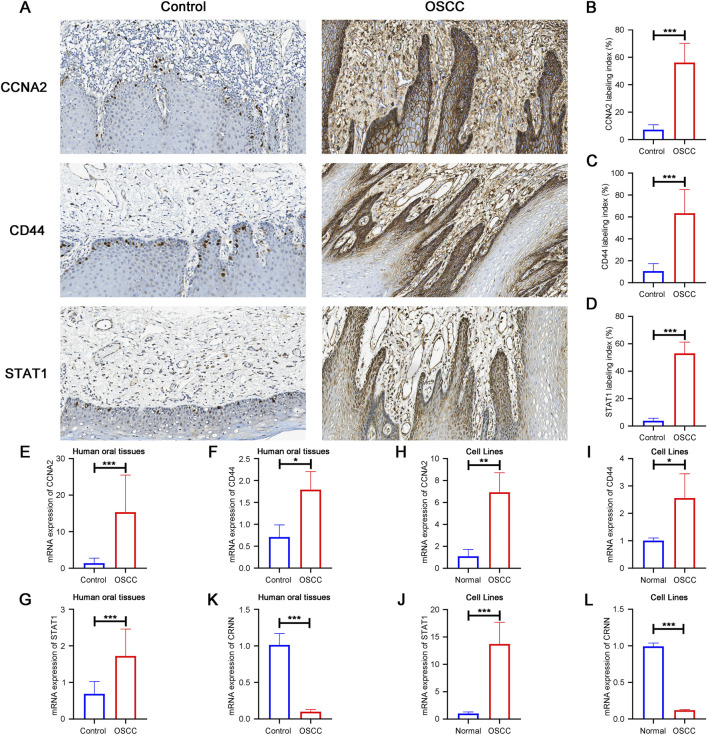
Experimental validation of CCNA2, CD44 and STAT1 expression in OSCC tissues and cell lines. **(A)** Representative IHC staining of CCNA2, CD44 and STAT1 in control and OSCC tissues. **(B–D)** Quantification of IHC staining index for CCNA2, CD44 and STAT1 in human oral tissues. **(E–G)** qPCR analysis of CCNA2, CD44 and STAT1 mRNA levels in human oral tissues. **(H–J)** qPCR analysis of CCNA2, CD44 and STAT1 mRNA levels in normal versus OSCC cell lines. **(K,L)** qPCR analysis of CRNN mRNA expression in human oral tissues and cell lines. Data are presented as mean ± SD. *P < 0.05, **P < 0.01, ***P < 0.001.

To experimentally validate the transcriptome-based findings, we assessed the protein and mRNA expression of CCNA2, CD44 and STAT1 in human OSCC tissues and cell models. Immunohistochemical staining revealed markedly stronger signals for all three markers in OSCC tissues compared with normal oral mucosa (Figure 5A), and semi-quantitative scoring confirmed significant upregulation (Figures 5B–D). Consistent with protein-level changes, qPCR analysis of human tissues demonstrated significantly elevated mRNA expression of all three genes in OSCC samples relative to controls (Figures 5E–G).

At the cellular level, qPCR analysis further verified that CAL27 cells expressed substantially higher levels of CCNA2, CD44 and STAT1 than normal oral epithelial cells (HOK) (Figures 5H–J). In addition to these upregulated hub genes, CRNN was selected as a representative downregulated gene based on the integrated transcriptomic analysis and further validated by qPCR. As shown in Figures 5K,L, CRNN expression was significantly reduced in OSCC tissues and cell lines compared with their corresponding controls. Together, these convergent findings across transcriptomic datasets, clinical specimens and cell models provide robust experimental evidence supporting the dysregulated expression patterns of key OSCC-associated genes. Consistent with the IHC findings, Western blot analysis demonstrated significantly increased total STAT1 protein levels in OSCC tissues and CAL27 cells relative to normal oral tissues and HOK cells, respectively (Supplementary Figure S3).

## 4 Discussion

OSCC is characterized by extensive transcriptional reprogramming, sustained proliferative signaling, epithelial remodeling, immune microenvironmental perturbation and genomic instability (Zhi et al., 2024; da Silva Junior et al., 2018; Yang et al., 2022; Truchard et al., 2022). In this study, we integrated multi-cohort transcriptomic analysis, network-based gene prioritization, outcome interrogation and experimental validation to identify OSCC-associated hub genes with cross-platform stability. Cross-dataset comparison revealed a reproducible transcriptional divergence between OSCC and normal tissues, and network-level inference converged on CCNA2, CD44 and STAT1 as central regulatory nodes with validated expression patterns and prognostic relevance. Functional enrichment suggested that these genes are embedded within proliferation-, adhesion- and stress-associated programs that are mechanistically aligned with OSCC biology. Their consistent elevation across public datasets, human tissues and cell models supports dual value for molecular characterization and translational applicability.

CCNA2, CD44 and STAT1 represent three biologically distinct yet functionally relevant classes of regulators. CCNA2 encodes a core cyclin that orchestrates mitotic progression and has been repeatedly implicated in epithelial tumor proliferation (Li et al., 2021; Zhou et al., 2024). CD44 functions as a stemness- and adhesion-related surface receptor widely linked to invasion, migration and metastatic competence across carcinomas (Senbanjo and Chellaiah, 2017; Xu et al., 2015; Li et al., 2022). STAT1 serves as a transcriptional effector of interferon signaling and has been reported to exert immune-mediated tumor restraint in a context-dependent manner (Koromilas and Sexl, 2013; Zimmerer et al., 2007). Given the immune-related function of STAT1, we further explored its association with the tumor immune microenvironment. Immune infiltration analyses suggested that STAT1 expression was positively associated with immune and stromal enrichment, indicating that its upregulation may partially reflect immune cell infiltration rather than tumor-intrinsic expression alone. This context-dependent feature may help explain the relatively favorable prognostic trend observed for STAT1 in OSCC. In OSCC, we observed that CCNA2 and CD44 were consistently upregulated and associated with adverse survival, consistent with their reported oncogenic behavior in epithelial malignancies (Zhang et al., 2024; Hassn et al., 2021). By contrast, STAT1 elevation correlated with more favorable prognosis and enrichment in immune- and stress-related pathways, aligning with literature describing STAT1-mediated antitumor surveillance under specific biological contexts (Zhang et al., 2020). Importantly, multivariate Cox proportional hazards analysis incorporating age, sex and tumor stage indicated that the prognostic associations of CCNA2, CD44 and STAT1 were attenuated after adjustment for established clinicopathological variables (Supplementary Table S2). While CD44 retained a marginal association with overall survival, CCNA2 and STAT1 did not demonstrate independent prognostic significance in the fully adjusted model. These findings suggest that the survival relevance of these hub genes may be partially mediated through their linkage with underlying tumor biology and clinical features, underscoring their complementary rather than standalone prognostic value.

GSEA-based enrichment provides mechanistic support linking the three hub genes to OSCC biology rather than statistical coincidence. CCNA2 enrichment in cell cycle and p53-associated programs is consistent with previous evidence showing that checkpoint override and mitotic drive are prerequisites for malignant expansion in epithelial cancers (Fang et al., 2022; Ruan et al., 2017). CD44 enrichment in tight-junction disruption, cancer-associated signaling and JAK–STAT activation aligns with reports that epithelial disassembly, ECM engagement and cytokine-integrated signaling are hallmarks of invasive transition in OSCC and related squamous tumors (Okamoto et al., 2016; Dolatabadi et al., 2022; Ortiz et al., 2018). STAT1 enrichment in interferon-, p53- and TGF-β–related circuits is consistent with its documented role in immune-linked stress restraint and tumor surveillance (Liang et al., 2004; Townsend et al., 2004). Of note, all three genes converged on p53 signaling and ubiquitin-mediated proteolysis, supporting the view that OSCC evolution arises not from a single linear oncogenic axis, but from sustained erosion of genome-stability surveillance and proteostasis control.

Although CCNA2, CD44 and STAT1 were validated at both the transcript and protein levels, the present study is primarily associative in design and does not establish that these genes are directly causative for OSCC progression. Definitive mechanistic involvement will require future functional perturbation studies, such as CRISPR-based gene disruption, rescue experiments or pharmacologic modulation.

With respect to STAT1, although its expression was consistently elevated and clinically associated, the current study focused on expression-level and network-associated evidence rather than post-translational activation status. In particular, phosphorylation at Tyr701 and Ser727, which is critical for STAT1 transcriptional activity, was not assessed in the present work. Accordingly, our findings should be interpreted as indicative of STAT1 dysregulation at the expression and network level, rather than direct confirmation of STAT1 signaling activation.

Moreover, all transcriptomic analyses in this study were conducted using bulk-tissue datasets, which limit the resolution of cell-type–specific expression patterns. As a result, the observed upregulation cannot be unequivocally attributed to malignant epithelial cells, infiltrating immune populations or stromal components. Future integration of single-cell or spatially resolved transcriptomic approaches will be necessary to clarify cellular provenance and regulatory context, thereby refining the biological interpretation of these findings.

Taken together, this study delineates a reproducible molecular axis in OSCC through integrated transcriptomic screening, network-level inference, multi-cohort validation and experimental confirmation. CCNA2, CD44 and STAT1 emerge not as incidental correlates but as network-defined and functionally anchored hub genes embedded in biological programs consistent with established determinants of OSCC evolution. Their consistent upregulation across independent datasets, human specimens and cell models, together with their diagnostic and prognostic relevance, positions this three-gene set as a rational molecular substrate for both mechanistic dissection and biomarker development. These findings refine the molecular landscape of OSCC and provide a defined entry point for subsequent functional, diagnostic and translational research.

## 5 Conclusion

This study identifies CCNA2, CD44 and STAT1 as reproducible and biologically associated hub genes in oral squamous cell carcinoma through the integration of multi-cohort transcriptomic analysis, network-level prioritization, clinical validation and experimental confirmation. These genes are embedded within proliferation-, adhesion- and stress-related signaling programs consistent with established pathogenic features of OSCC, and they exhibit consistent upregulation across independent datasets, human specimens and cell models. The diagnostic and prognostic relevance of this three-gene set, together with its network positioning, provides a rational molecular basis for downstream mechanistic interrogation and biomarker development. Overall, these findings refine the molecular framework of OSCC and establish CCNA2, CD44 and STAT1 as promising candidates for future functional and translational research.

## Data Availability

The transcriptomic data analyzed in this study are publicly available in the NCBI Gene Expression Omnibus (GEO) repository under accession numbers GSE28133 and GSE285957. All scripts used for data preprocessing and analysis are available at https://github.com/liujiangying68/PVR-ferroptosis-transcriptomics
