# Supplementary material for: Integrated network analysis and experimental validation identify CCNA2, CD44, and STAT1 as clinically relevant hub genes in oral squamous cell carcinoma
Source: Front Mol Biosci. 2026 Feb 9;13:1748821. doi: 10.3389/fmolb.2026.1748821 (PMC12926659; doi:10.3389/fmolb.2026.1748821)
Supplement: Supplementary file 1 [file Supplementaryfile1.docx]

Supplementary Material


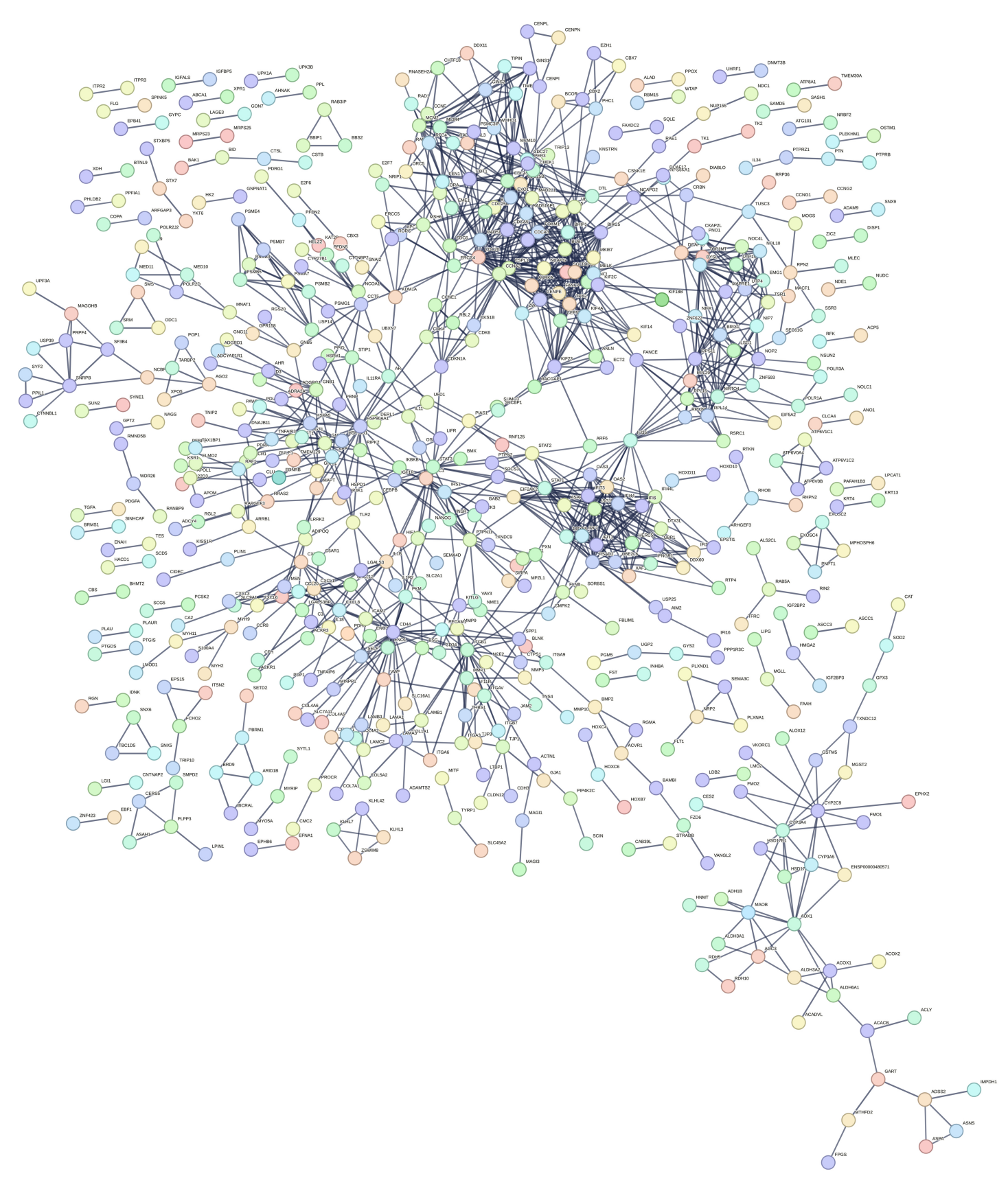
**Supplementary Figure 1.** PPI network diagram of overlapping genes constructed with STRING.


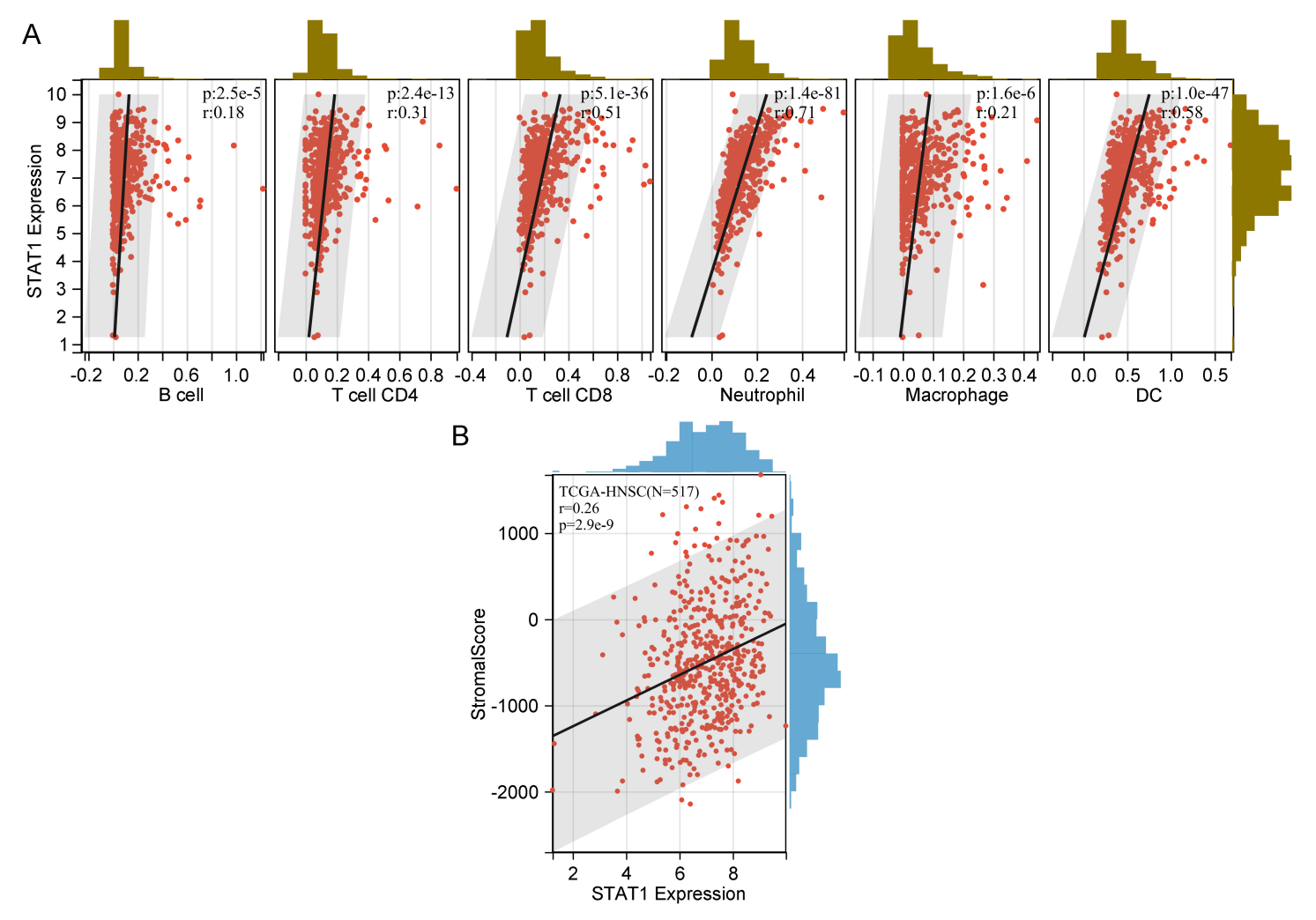


**Supplementary Figure 2.** (A) Correlations between STAT1 expression and estimated infiltration levels of B cells, CD4⁺ T cells, CD8⁺ T cells, neutrophils, macrophages and dendritic cells in the TCGA-HNSC cohort based on the TIMER algorithm. (B) Correlation between STAT1 expression and stromal score calculated using the ESTIMATE algorithm in TCGA-HNSC samples.





**Supplementary Figure 3.** Validation of total STAT1 protein expression in OSCC. (A) Representative Western blot images showing total STAT1 protein expression in human normal oral tissues and OSCC tissues, with β-actin used as a loading control. (B) Quantitative analysis of STAT1 protein levels normalized to β-actin in human oral tissues (n = 6 per group). (C) Representative Western blot images of total STAT1 protein expression in normal oral epithelial cells (HOK) and OSCC cell line (CAL27). (D) Densitometric quantification of STAT1 protein expression in cell lines, normalized to β-actin and expressed relative to HOK. Data are presented as mean ± SD. **P < 0.01, *P < 0.001.





**Supplementary Table 1.** Sensitivity analysis of hub gene identification across different network centrality thresholds. Hub genes were ranked using degree and betweenness centrality metrics under varying cutoff thresholds (top 5, top 10 and top 15 nodes). The overlap of candidate genes across thresholds demonstrates the robustness and stability of CCNA2, CD44 and STAT1 as central network nodes in OSCC-associated protein–protein interaction networks.





**Supplementary Table 2.** Univariate and multivariate Cox regression analysis of CCNA2, CD44, STAT1 and clinicopathological variables in TCGA OSCC cohort.
